# Immunotherapy Plus Cryotherapy: Potential Augmented Abscopal Effect for Advanced Cancers

**DOI:** 10.3389/fonc.2018.00085

**Published:** 2018-03-28

**Authors:** Joe Abdo, David L. Cornell, Sumeet K. Mittal, Devendra K. Agrawal

**Affiliations:** ^1^Department of Clinical and Translational Science, Creighton University School of Medicine, Omaha, NE, United States; ^2^Department of Surgery, CHI Health Creighton University Medical Center, Omaha, NE, United States; ^3^Dignity Health, Norton Thoracic Institute, St. Joseph’s Hospital and Medical Center, Phoenix, AZ, United States

**Keywords:** abscopal effect, immunotherapy, anti-PD1, anti-CTLA-4, cryoablation, self-antigens, autoinoculation, cancer immunity

## Abstract

Since the 1920s the gold standard for treating cancer has been surgery, which is typically preceded or followed with chemotherapy and/or radiation, a process that perhaps contributes to the destruction of a patient’s immune defense system. Cryosurgery ablation of a solid tumor is mechanistically similar to a vaccination where hundreds of unique antigens from a heterogeneous population of tumor cells derived from the invading cancer are released. However, releasing tumor-derived self-antigens into circulation may not be sufficient enough to overcome the checkpoint escape mechanisms some cancers have evolved to avoid immune responses. The potentiated immune response caused by blocking tumor checkpoints designed to prevent programmed cell death may be the optimal treatment method for the immune system to recognize these new circulating cryoablated self-antigens. Preclinical and clinical evidence exists for the complementary roles for Cytotoxic T-lymphocyte-associated protein (CTLA-4) and PD-1 antagonists in regulating adaptive immunity, demonstrating that combination immunotherapy followed by cryosurgery provides a more targeted immune response to distant lesions, a phenomenon known as the abscopal effect. We propose that when the host’s immune system has been “primed” with combined anti-CTLA-4 and anti-PD-1 adjuvants prior to cryosurgery, the preserved cryoablated tumor antigens will be presented and processed by the host’s immune system resulting in a robust cytotoxic CD8^+^ T-cell response. Based on recent investigations and well-described biochemical mechanisms presented herein, a polyvalent autoinoculation of many tumor-specific antigens, derived from a heterogeneous population of tumor cancer cells, would present to an unhindered yet pre-sensitized immune system yielding a superior advantage in locating, recognizing, and destroying tumor cells throughout the body.

## Introduction

In the era of molecular medicine, the oncology field still faces many extreme challenges extending survival rates to all stage III and IV cancers with many cancers remaining particularly arduous to treat. Five-year survival rates for lung, liver, and esophageal cancers have hovered around only 15% for decades (1). Other diseases, such as mesothelioma, pancreatic carcinoma, and glioblastomas, have only single digit 5-year survival rates (1). All cancers with distant metastases have less than a 10% 5-year survival. For many of these difficult challenges, new approaches to extend survival are under constant clinical investigation. Herein, we propose a novel approach combining two dependable oncology-based treatment platforms to potentially cause a synergistic immune response to target loco-regional and distant cancer metastases efficiently.

**Figure 1:**
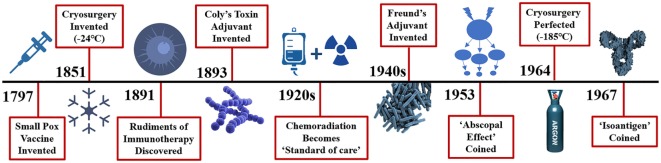
Distant history of the rudiments of immunotherapy and cryosurgery. Efforts to stimulate or potentiate the immune response to diseases date back a millenium with the Greeks in fifth century BC attempting immunotherapy to combat the plague and ancient Chinese medical innovations to combat smallpox in 1000 AD. More recently, immune adjuvants such as Coly’s toxin in the 1890s and Freund’s adjuvant introduced in the 1940s yielded remarkable tumor regression. Surprisingly, injection of peri-tumor streptococcal infection results in a 39% reduction in cancer. Why further investigations of these adjuvants have fallen out of favor is not clear, however, new approaches in the effort to eradicate cancer took a turn to newer agents including chemotherapy, and radiation therapy, either in serially or parallel fashion along with hormonal adjuncts. The abscopal effect is defined as: the regression of distant metastases following treatment of the primary cancer. The first definitive observed abscopal effect was seen with radiation treatment of brain metastases in 1953. The abscopal effect was described in cryosurgery of an advance prostate cancer in 1963. In 1967 Yantorno et al. first identified and described how cryosurgical ablative intervention resulted in the production of antibodies directed at antigenic material linked to the frozen tissue itself. They reported that freezing male rabbit accessory tissue which was sensitized with glandular cellular components *via* injection led to the formation of circulating antibodies with a specified target.

Cryosurgery was first mentioned in the papyrus papers around 3000 BC when Egyptians and then Hippocrates used it to treat skull fractures and infected wounds (2). Napoleon’s legendary surgeon, Dominique-Jean Larrey, used it to facilitate amputations during his historic retreat from Moscow between 1845 and 1851 (2). Modern cryosurgery techniques were then used in 1850 when James Arnott described “destroying the vitality of the cancer cell” utilizing an iced salt solution that reached −24°C to treat breast and uterine cancers (3). Various methods and techniques evolved over the next century, but it was not until Cooper and Lee developed a liquid nitrogen-based cryosurgical apparatus to freeze tumors that this platform found its clinical utility (4). Cooper then used cryosurgery to freeze the thalamic tract as a treatment for tremors in Parkinson’s disease (5). Gage and Gonder et al. (6) developed a modified cryosurgical device to freeze prostatic cancers transurethrally in 1964 which propelled the modern cryosurgery era (Figure 1). Applying the Joule-Thomson effect in an experimental argon gas system, Torre achieved temperatures down to −185°C (7). This allowed engineers utilizing thermo-coupling of pressurized gases and cooling to develop smaller instruments and thin needle-like probes for the cryosurgical destruction of various tissues and tumors. Finally, Onik et al. (8) utilized ultrasound to refine cryosurgery with monitoring of the “cryolesion,” more commonly referred to as the “ice ball.” These advancements led to the renaissance of cryosurgery in the 1990s. Currently, cryosurgery applications are broad and wide, varying from treating brain tumors and lung cancers to metastases of liver and lung from a variety of primary cancer sites, to breast adenomas and cancers, melanoma and skin cancers to name a few (9).

A series of papers by Shulman, Yantorno, Soanes, and Gonder from 1965–1967 illustrated how antibodies elicited by cryosurgery of the prostate gland and accessory tissues release circulating antigens and referred to this process as cryo-immunization and coined the term iso-antigens or self-antigens (10). Soanes, Ablin, and Gonder then published the first case report of three human prostate cancer patients who demonstrated the cryosurgical abscopal effect: regression of distant metastases including lesions of the cervical spine, pulmonary metastases, and left supraclavicular lymph nodes metastases following cryoablation of the primary prostate cancer (11). In the following years, investigations aimed to elucidate the mechanisms and subsequent effect of the immunologic response to cryosurgery. Scientists revealed the clinical benefit of cancer antigens, which stimulate the production of antitumor antibodies, cytotoxic T-cells and produce a robust cytokine response targeted specifically toward malignant cells (12). Review of the literature reveals that there is a highly variable immune response to cryosurgery that is stimulatory or suppressive, representing a finely tuned and orchestrated series of events reaching a homeostatic point between the adaptive and innate immune responses. Manipulating this variable response to favor a more cytotoxic immune response would be highly advantageous.

However, releasing self-antigens into circulation may not be sufficient to overcome the escape mechanisms and checkpoints many cancers have evolved to escape detection and the host’s subsequent immune response. Altering the host’s immune system by blocking these checkpoints designed to prevent programmed cell death may be instrumental in allowing the immune system to recognize these new cryoablated circulating self-antigens, thereby potentially causing a robust immune response to kill distant metastases; a phenomenon known as the abscopal effect. Optimizing the immune response is, therefore, essential to conquering stage III and IV cancers. Enhancing the antigenic immune response to cryosurgery would then seem to be an ideal avenue to promote a cryosurgery-based abscopal effect.

Today modern cryosurgery is exclusively used to treat a spectrum of tumors and cancers with various indications from benign adenomas and precancerous lesions to cancer lesions and low-grade or localized early solid tumors. Cryosurgery is also used to treat metastases to the liver and lungs and other organs when complete surgical extirpation is not an option or merely as a way to slow down progression of the disease (metastatic) process through debulking. However, there are no standard patient treatment protocols calling for the use of cryotherapy in any later stage III or IV cancers. Some indications for the use of cryotherapy for treating early cancers include the eradication of small retinoblastomas, basal and squamous cell skin cancers, cervical intraepithelial neoplasia, and low-grade bone tumors. Other benefits of cryosurgery in early stage breast, liver, prostate, colon, kidney, pancreatic, and esophageal cancers are currently under investigation (Table 1). The FACT, FROST, ECLIPSE, and SOLSTICE Studies and other additional human trials of cryoablation in benign and early stage/resectable breast, lung and esophageal cancers are in progress (9).

**Table 1:** Pioneering cryotherapy studies in the oncology arena.

Study	Indication	Results	Reference	Year
FACT—FibroAdenoma Cryoablation Treatment Registry	Benign breast fibro adenoma	For patients with a fibroadenoma smaller than 2 cm, complete resolution can be expected in 66% of patients at 6 months and 75% of patients at 12 months, respectively	Nurko et al. (13)	2005

Percutaneous ultrasonography-guided pancreatic cryoablation: feasibility and safety assessment	Stage II–IV pancreatic cancer	Mean and median survival was 15.9 and 12.6 months, respectively. The 6-, 12-, and 24-month survival rates were 82.8, 54.7, and 27.3%. 27 out of 32 patients experienced a ≥50% reduction in pain score	Niu et al. (14)	2012

ECLIPSE—Evaluating Cryoablation of Metastatic Lung Tumors in Patients	Colon (40%), Kidney (23%), and Sarcomas (8%)	Local tumor control rates were 56 of 58 (96.6%) and 49 of 52 (94.2%) at 6 and 12 months, respectively. 1-year overall survival rate was 97.5%	de Baere et al. (9)	2015

CT guided cryoablation for metastatic bone and soft tissue tumors	Metastatic bone and soft tissue tumors	At the final follow up, 4 of 9 patients showed no evidence of disease, 2 of 9 were alive with disease, and 3 of 9 patients died of disease	Susa et al. (15)	2016

A Phase II Trial of Cryoablation Therapy in the Treatment of Invasive Breast Carcinoma	Early stage breast cancer	Successful ablation occurred in 80/87 (92%) of cancers	Simmons et al. (16)	2016

Safety and efficacy of endoscopic cryotherapy for esophageal cancer.	Early stage esophageal adenocarcinoma	86 patients completed treatment with complete response of intraluminal disease in 55.8%, including complete response in 76.3% for T1a, 45.8% for T1b, 66.2% for all T1, and 6.7% for T2. Mean follow-up was 18.4 months	Tsai et al. (17)	2017

Cryoablation w/natural killer cell therapy and Herceptin in recurrent breast cancer patients	HER2-overexpressing breast cancer	43.75% of patients treated with all three modalities achieved partial regression and 31.25% patients exhibited stable disease. Only 2 of 16 (12.5%) patients exhibited progressive disease. 3 of 16 patients had complete regression	Liang et al. (18)	2017

Cryo-Assisted Resection En Bloc, and Cryoablation *In Situ*, of Primary Breast Cancer	Breast cancer	The frozen site extruded the dye that was distributed through the unfrozen tumor, the breast tissue, and the resection cavity for 12 of 14 patients	Korpan et al. (19)	2018

SOLSTICE—Study of Cryoablation for Metastatic Lung Tumors	Neoplasm Metastasis	Evaluate the safety and efficacy of cryoablation therapy in patients with pulmonary metastatic disease. Patients will undergo cryoablation of at least 1 metastatic pulmonary tumor and will be followed 24 months post cryoablation	Callstrom, M	2019

Pembrolizumab and Cryosurgery in Treating Newly Diagnosed Prostate Cancer	Oligo-metastatic Prostate Cancer	Phase II trial analyzing the safety and efficacy of pembrolizumab and cryosurgery along with short term androgen ablation to treat patients with oligo-metastatic prostate cancer	Ross, A	2019

trūFreeze^®^ Spray Cryotherapy Patient Registry	Esophageal adenocarcinoma/Barrett’s esophagus	Currently collecting efficacy and outcomes data related to the use of trūFreeze^®^ spray cryotherapy for the treatment of unwanted tissue in the pulmonary and gastrointestinal settings	Shaheen, N	2020

FROST—Cryoablation of Small Breast Tumors in Early Stage Breast Cancer	Breast Cancer	The hypothesis is that cryoablation will completely destroy early stage invasive breast cancer tumors in a selected population of women who may otherwise be adequately treated with surgery. No results yet	Holmes, D	2021

*Results of clinical trials and preclinical studies attempting to determine the safety and efficacy of cryoablation therapy in a myriad of cancers and stages. Overall, cryotherapy is deemed a safe and efficacious method for primary tumor destruction as well as efficient in eradicating metastatic lung lesions as a palliative measure. Large, multicenter clinical trials are also ongoing with the details included herein (9, 13–19)*.

Our hypothesis, however, is not interested in the physical mechanisms involved when cryosurgery causes a reduction in the tumor size of early or later stage cancers, but rather the immunological effect induced by the freezing and breaking of tumors, potentially rescuing a patient whose disease has spread to nearby regional lymph nodes (stage III) or other distant organs (stage IV). Hence, our focus here is on how the combination of cryotherapy, preceded by dual immunotherapy, could result in regression of disease in later stage III and IV cancers. This approach would be a novel use of cryotherapy as it is currently used today to either extirpate or debulk cancers without consideration of its potentially more powerful effect on the progression of the cancer and/or its inhibition of metastatic spread to distant organs.

The immune system is comprised of the innate and adaptive immune responses and is a highly complex and orchestrated system between these two arms. The adaptive response controls the development of targeted cytotoxicity and anti-cancer responses with long term immunologic memory, a process that is the basis of vaccination. Antigens are either exogenous or endogenous intracellular antigens. Intracellular antigens are processed by MHC class I molecules which activate cytotoxic CD8^+^ T-cells (20). Cytotoxic T-cells are comprised of either CD4^+^ helper cells or CD8^+^ cytotoxic T-lymphocytes (20). Exogenous antigens are typically expressed on cells that exhibit MHC class II molecules whereas intracellular or endogenous antigens are expressed by cells with MHC class I molecules (21). How these antigens are presented and processed, and the subsequent balance and homeostasis set-point of the MHC Class cellular response and their cytokine profile that develops will dictate the T-cell and cytotoxic response which ultimately results in destruction and killing of the invading organism or cancer cell senescence (22). Ideally, a T_H_1 response would favor eradication of cancer *via* the classic dendritic cell presentation of intracellular viral or self-components resulting in abrogation of “self” cancer cells (23). However, the host must control this response to prevent rejection of self, and this often results in a suppressive response to malignant cells with the cancer once again evading detection.

Altering or changing this balance of MHC class presentation will subsequently change the T-cell response. Cryosurgery likely alters this antigen presentation and/or cytokine profile which may then result in a more favorable and robust MHC class I CD8^+^ cytotoxic anti-cancer response (24). T-regulatory cells (Tregs) are made of T-suppressor cell and T-effector cells and are typically recognized as CD4^+^CD25^+^FoxP3^+/−^ (25, 26). The balance and ratio of T-suppressor cells to T-effector cells may be the responsible shift that occurs causing a change to the more favorable CD8^+^ cytotoxic T-cells (27). This response can be dampened or inhibited if the antigens presented by cytotoxic T-cells are not promoted. Antigen-presenting cells (APCs) interact and recognize self through the CD28/B7 receptors on APCs and T-cells, respectively, and through their respective cytokine profile promote further production of the cellular response (28). CD28 transmits a stimulatory signal promoting T-cell propagation (28). Cytotoxic T-lymphocyte-associated protein (CTLA-4) is constitutively expressed on Tregs but is only expressed on T-cells once activated (29). CTLA-4 binds to the exact same receptors as CD28 but more avidly and is an inhibitory signal thought to dampen and prevent an auto-immune response and promote self-tolerance (24). CTLA-4 inhibits over production or prevents an over-zealous T-cell response. This inhibitory signal is notable in many cancers and those cancers promote the development of these inhibitory molecules as escape mechanisms to prevent the further production of CD8^+^ cytotoxic T-cells (29). Another well-described inhibitory molecule/receptor that cancers have developed as an escape mechanism is PD-1 and its ligand, PD-L1. These inhibitory receptors and ligands are known as immune checkpoints and are again, inhibitory. Cancers utilize these immune checkpoints as an escape mechanism by binding to the CTLA-4 and PD receptors more competitively and stronger, thus inhibiting the cytotoxic T-Cell and immune response and avoiding detection and/or destruction by the immune system (30).

The innate and adaptive immune system crosstalk is controlled by Tregs. Tregs modulate and control the homeostatic “set point” to prevent autoimmune diseases and balance the systemic inflammatory response (31). The ratio of Tregs to T-effector cells and their subsequent cytokine profile dictate and control this “set-point,” representing the most efficient and safest mechanism to eradicate the invading organism while protecting the host. This balance is necessary, as an exaggerated response can occur which may result in autoimmune disease or processes such as persistent inflammatory compensatory syndrome; resulting in damage to the patient’s physiology.

Cytotoxic T-lymphocyte-associated protein is a transmembrane cell receptor expressed on the surface of activated T-cells (32). CTLA-4 is not expressed on resting T-cells; however, they are present on Tregs which function by upholding tolerance to self-antigens (32). CTLA-4 possesses homology to CD28 and can be found on activated CD4^+^ and CD8^+^ T-cells subsequent to T-cell receptor induction (33). A highly active T-cell co-stimulatory signal is controlled by CD28 which binds to B7-1 (CD80) and B7-2 (CD86) ligands on APCs and induces T-cell proliferation by generating expression of interleukin-2 (IL-2) and a cluster of anti-apoptotic factors (34). CTLA-4 has greater affinity and avidity to B7-1 and B7-2 compared to CD28 (34). Therefore, minimal amount of CTLA-4 effectively out-competes CD28 ligand binding and attenuates the T-cell response as CTLA-4 is inhibitory whereas CD28 binding stimulates the T-cell response.

PD-1 is also a negative, costimulatory protein that is located primarily on the surface of activated CD4^+^ and CD8^+^ T-cells (28). Signaling through PD-1 inhibits the capability of T-cells to proliferate and produce cytokines and attenuates cytotoxic T-cell function. PD-L1 is exclusively expressed on the surface of tumor cells and is the ligand that binds with PD-1 which causes programmed cell death to be turned off (35). Like CTLA-4, the PD-1 pathway down-modulates T-cell activation by turning on overlying signaling molecules of the immune checkpoint pathway (36). CTLA-4 and PD-1 are frequently expressed on regulatory T-cells irrespective of PD-L1 tumor expression levels. CTLA-4 and PD-1 function represent two separate regulatory pathways that mutually intersect with the immune system checkpoint inhibition. When a patient is not afflicted with cancer, these biomarkers are crucial in maintaining self-tolerance. When a patient has cancer, the disease will bind to these receptors more avidly and subsequently allow the progression and spread of the cancer. Conversely, blockade of these checkpoint pathways enhances the immune response to cancers such as non-small cell lung carcinoma, renal cell carcinoma and melanoma and potentially other cancers as well (36).

**Figure 2:**
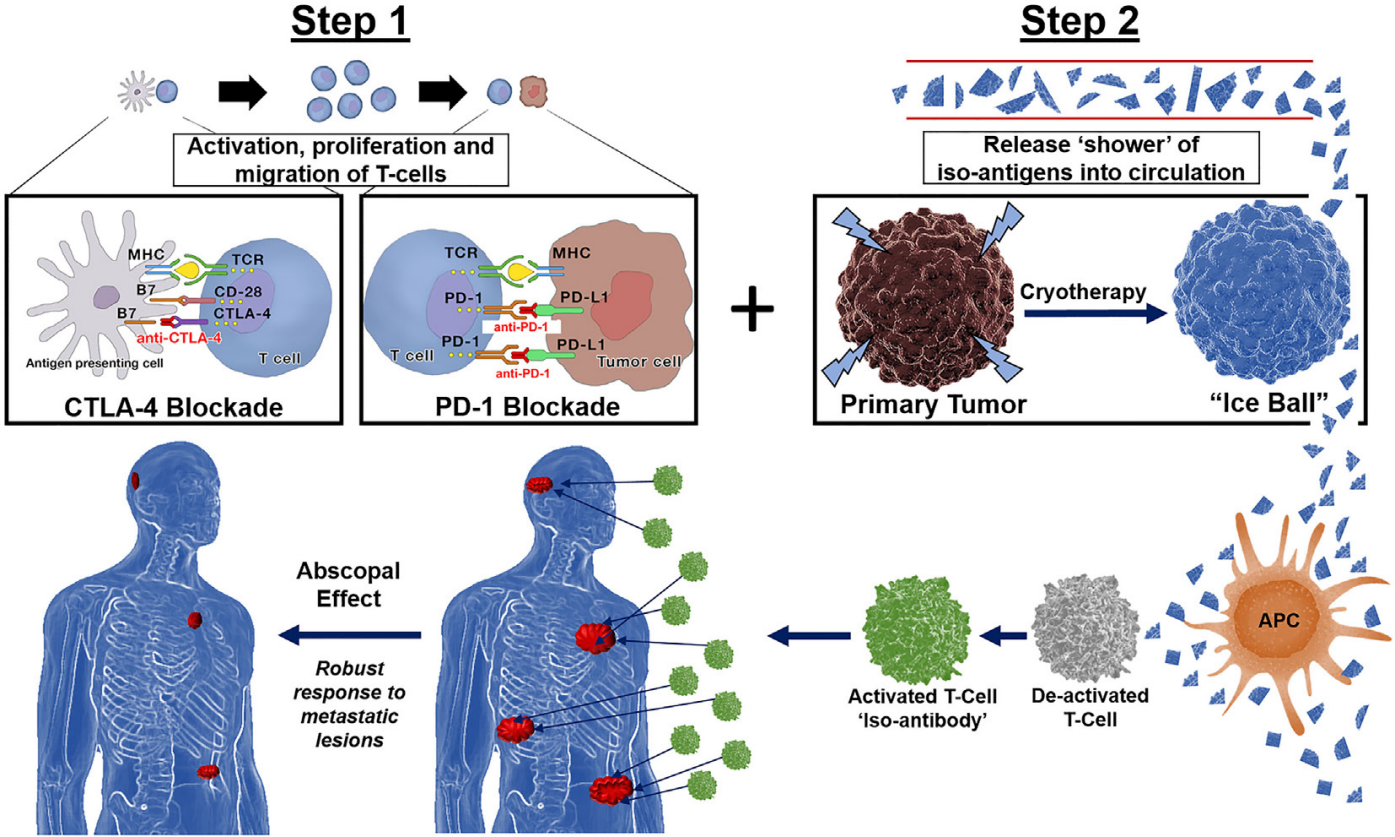
Immunotherapy followed by cryotherapy for enhanced abscopal effect: If polyvalent, auto-inoculating tumor vaccines can be augmented and potentiated with the combined checkpoint inhibitors cytotoxic T-lymphocyte-associated protein (CTLA-4) and PD-1, then theoretically, a robust abscopal effect should be observed within the host, not only by cryosurgery tumor ablation/destruction of the primary lesion but also by abrogation of regional and distant metastases along with conferred immunity at both local and distant tumor sites. Hence, through synergistic processes these checkpoint inhibitors exert their effects on the immune processing of a large polyvalent vaccine caused by cryosurgical destruction and release of a large population of heterogenous tumor antigens; the hosts immune system should respond accordingly as is currently being witnessed and reported in the literature today *via* the very same processes encountered with radiation oncology in combination with these novel checkpoint inhibitors CTLA-4 and PD-1, yielding a robust cryosurgery-based abscopal effect.

Preclinical and clinical evidence exist for the complementary roles for PD-1 and CTLA-4 antagonists in influencing adaptive immunity, showing evidence that combination immunotherapy provides for a more robust response to distant lesions (37). Co-stimulating the immune system with a shower of tumor antigens *via* cryosurgery in the presence of a “primed” immune system pre-treated with CTLA-4 and PD-1 combined therapy would theoretically result in a synergistic effect of local tumor and distant metastases regression, or abscopal effect (Figure 2).

The abscopal effect was identified over 50 years ago and refers to the phenomenon following radiation therapy of a primary tumor which results in regression of distant metastatic lesions. This, presumably is due to the release of tumor-specific iso(self)-antigens into the patient’s circulation and the immune response that manifests thereafter. Indeed, there now exists numerous reports in the literature where the abscopal effect has been observed when patients who were previously treated with CTLA-4 and PD-1 antibodies subsequently received radiation therapy, and their distant metastases regressed (Table 2) (38). This abscopal effect is believed to be mediated by the development of a systemic antitumor immune response caused by radiation therapy, which releases intratumoral antigens adjacent to APCs and T-cells; quite similar if not identical to the immune response resultant of cryoablation. Notably, cryoablation has less adverse events associated with its use compared to radiation and may provide for a more comprehensive and efficient release of self-antigens into circulation (39–41). Cryosurgery likely preserves these self-antigens whereas radiation, radiofrequency, and microwave therapy denatures and destroys some of these circulating tumor epitopes (42).

**Table 2:** Clinical trials that have yielded robust abscopal effects.

Study	Indication	Results	First Author	Year
Clinical response to cellular immunotherapy and intensity-modulated radiation	Advanced solid tumor cancers	– Complete responses were achieved in 34 of 58 of recurrent lesions. – Partial responses were achieved in 17 of 58 patients. – Patients manifested an increase in CD8^+^CD56^+^ lymphocytes.	Hasumi et al. (43)	2013

Abscopal response to radiation and anti-CTLA-4 therapy	Metastatic non-small cell lung adenocarcinoma	– Reported the first abscopal response in a treatment-refractory lung cancer. – Increase in tumor-infiltrating cytotoxic lymphocytes. – Tumor regression observed. 1 year after treatment patient has no evidence of disease.	Golden et al. (44)	2013

Phase II: anti-CTLA-4 alone or in combination with radiotherapy	Metastatic castration-resistant prostate cancer	– Among patients receiving 10 mg/kg ± radiotherapy, 8 had PSA declines of ≥50% (duration: 3–13 + months), one had complete response (duration: 11.3 + months), and 6 had stable disease (duration: 2.8–6.1 months).	Slovin et al. (45)	2013

Abscopal effect of anti-CTLA-4 and radiation therapy	Melanoma with brain metastases	– Patients who received ipilimumab had a median survival of 18.3 months, compared with 5.3 months. 40% of ipilimumab patients had a partial response compared to 9.1% of patients who just received radiation.	Silk et al. (46)	2013

Abscopal effect of radiation and immunotherapy	Stage IV melanoma	– All metastases, including unirradiated liver lesions had completely resolved, consistent with a complete response.	Hiniker et al. (47)	2012

Abscopal effect of anti-CTLA-4 therapy in melanoma (NEJM)	Stage III and IV melanoma	– Tumor shrinkage with specific antibody responses to the cancer. – Changes in peripheral-blood immune cells observed. – Increases in antibody responses to other antigens after radiotherapy.	Postow et al. (48)	2012

External beam radiotherapy with intratumoral injection of dendritic cells	Soft Tissue Sarcoma in neoadjuvant setting	– 9 of 17 patients developed tumor-specific immune responses (11–42 weeks). – 12 of 17 patients were progression free after 1 year. – Treatment caused a dramatic accumulation of T-cells in the tumor.	Finkelstein et al (49)	2012

Phase II: *in situ* vaccination by intratumoral TLR9 agonist combined with radiation	Low-grade B-cell lymphoma	– 5 of 15 patients had a complete response. – Immunized sites showed a significant reduction of CD25(+), Foxp3(+) T-cells and a similar reduction in S100(+), CD1a(+) dendritic cells.	Kim et al. (50)	2012

Phase I: stereotactic body radiotherapy and IL-2	Metastatic melanoma or renal cell carcinoma	– 8 of 12 patients achieved a complete or partial response. – 5 of 7 melanoma patients had a complete or partial response. – Greater frequency of proliferating CD4(+) T-cells in responding patients.	Seung et al. (51)	2012

Phase II: abscopal effect of anti-CTLA-4 therapy alone	Melanoma with brain metastases	– 14 out of 72 had a complete response in brain metastases. – 15 out of 72 patients exhibited disease control outside of the brain. – Anti-CTLA-4 was effective in small and asymptomatic metastases.	Margolin et al. (52)	2012

Abscopal effect associated with a systemic anti-melanoma immune response	Metastatic melanoma	– Serology showed anti-MAGEA3 antibodies, documenting an association between the abscopal effect and a systemic antitumor immune response. – The patient experienced a complete remission and resolution of nodal metastases.	Stamell et al. (53)	2013

Phase II: *in situ* vaccination with a TLR9 agonist induces systemic regression	Low-grade B-cell lymphoma	– 1 of 15 patients had a complete response. 3 of 15 had partial responses. – Some tumors induced a suppressive, regulatory phenotype in autologous T-cells *in vitro*; these patients had a shorter time to disease progression.	Brody et al. (54)	2010

Phase I: Coley’s toxins trial	Metastatic bladder cancer	– The toxins were effective at inducing fever and robust surges in cytokines. – Patient had a 50% reduction in his cancer.	Karbach et al. (55)	2012

Phase II: cancer vaccine with radiotherapy in prostate cancer	Local prostate cancer	– 13 of 17 patients had increases in PSA-specific T cells of at least 3-fold vs. no detectable increases in the radiotherapy-only arm (*P* < 0.0005). – Indirect evidence of immune-mediated tumor killing was evident.	Gulley et al. (56)	2005

Phase I: radiotherapy and intratumoral adoptive dendritic cell immunotherapy	Refractory hepatocellular carcinoma	– 10/12 patients had completed immunologic response. – AFP-specific immune response in 8/12 patients. – l6/12 patients showed increased NK cell cytotoxic activity post vaccination.	Chi et al. (57)	2005

*Results of clinical trials measuring the abscopal effect of radiotherapy combined with either immunotherapy, Coley’s toxins, or cancer vaccines—in the last decade. All 15 clinical trials produced positive results, verifying that regression of distant metastases is possible with destruction of the primary tumor after immune checkpoint blockade. We believe substituting radiation with cryoablation therapy would result in a more robust immune response and with a better safety profile for patients (43–57)*.

Unlike radiation therapy, cryoablation results in direct cell death by removing heat. Heat is transferred out of the tumor and surrounding tissues creating an inner zone of direct cell death *via* lethal hypothermia while causing indirect cell death in the outer zone *via* apoptotic signaling (58, 59). In the outer zone, where cells are not exposed to deadly below zero temperatures, injury to mitochondria results in delayed apoptotic cell-death. The ratio of apoptosis to necrosis following cryotherapy significantly influences the immunostimulating effect (60). Cryoablation induces a much greater post-intervention immune response compared to radiation therapy due to this ratio which can be observed by significantly increased levels of IL-1, IL-6, NFκB, and TNF-α post cryoablation (61–64). Furthermore, in comparative animal models, the amount of dendritic cell antigen loading is greater with cryoablation compared to radiation (65). Presumably, the degree of disparity in targeted immune activity is because radiation-based methods cause protein denaturation, decreasing the quantity of undamaged antigens released into circulation. Freezing alternatively preserves cellular ultrastructure and increases plasma membrane permeability. Cryoablation yields the discharge of intracellular debris, causing a release of cytokines that are associated with the systemic inflammatory response syndrome which is yet another indication of the robust immune response elicited by freezing primary cancer lesions (64). A similar phenomenon is not observed in heat or radiation-based modalities (66).

## Vaccines and Cancer

Historically, surgery has been and still is the gold standard for treating and extirpating a solid tumor. Chemotherapy and/or radiation therapy, whether neoadjuvant or adjuvant to surgery, are the key adjuncts to treat and cure cancer since the turn of the century, respectively (Figure 1). Although these adjuvants have helped to prolong disease-free survival and overall survival in many cancers, their adverse drug responses (ADRs) are well known and described (67). Over half of the ADRs are not preventable and many can be deleterious to the patient. Some of the more common ADRs are nausea, emesis, alopecia, diarrhea, fatigue, mucositis, myelosuppression, immunosuppression and sepsis (68). Sepsis is the one of the most feared ADRs in oncology with a 30% mortality rate, along with recent reports indicating that clinical sepsis impairs CD8 T-cell-mediated immunity (69, 70). One must, therefore, wonder what these deleterious effects have on cancer patient’s immune system. Have we perhaps inhibited the host’s own immune defense mechanism and aided the cancer in its detection and destruction due to the ADRs immunosuppressive effects? Although there is evidence that chemotherapy can potentiate the host’s immune system response, it would behoove us to consider alternative methods of cancer treatments that may not be so toxic to the host’s immune defense system but rather potentiate a targeted immune response (71, 72).

Vaccines are the most efficacious and only mechanism to completely eradicate a human disease. Diseases such as smallpox, measles, tuberculosis, and polio have been effectively eradicated by our immune response to vaccination. Once vaccinated, the host is protected from future insult and illness by the inciting virus, with the immune system pre-programmed to kill and destroy any virus resembling the original antigen. Gardasil^®^9, the vaccine for cervical cancer is administered to males and females to prevent both transmission and infection of the human papilloma virus (HPV) virus, ultimately preventing progression to cancer (73).

Numerous cancer vaccines have been developed but were fraught with failure in the recent past; such as Canvaxin™ for melanoma, MAGE-A3 for melanoma and lung cancer, OncoVAX^®^ for colon cancer, Sipuleucel-T (Provenge^®^) for prostate cancer, and Oncolytic vaccines for glioblastomas. Recently however, some of these and other cancer vaccines have been combined with immune checkpoint inhibitors with renewed interest and profound effects. Canvaxin™ is a therapeutic polyvalent cancer vaccine that almost achieved FDA approval in 2004. It is an antigen-rich, allogeneic vaccine derived from multiple melanoma cell lines in 1984 and is one of the most extensively analyzed cancer vaccines in oncology (74). Yet, in 2005 the Phase III clinical trial of Canvaxin™ in patients with advanced melanoma was terminated due to underwhelming performance (75). Now with the availability of checkpoint inhibitors, research and development for cancer vaccines like Canvaxin™ are making a resounding and successful return within the oncology field (76, 77). With these and other similar promising findings recently published, the NCI has granted millions of dollars in funding to conduct phase III clinical trials testing targeted vaccines combined with immune checkpoint inhibitors in patients with Stage III or Stage IV melanoma (78). This re-emerging platform has also been found to be efficacious in colon cancer. Thus phase II trials for late-stage colon cancer vaccines combined with immune checkpoint inhibitors are currently being developed (79). Remarkably, immunotherapy has reinvigorated research of cancer vaccines as a highly targeted treatment against cancer. It will be interesting to see if this combined approach becomes the future treatment mainstay in the clinical oncology arena.

We must, therefore, ask: Is there an anti-cancer treatment process that is analogous to a vaccine? We herein propose that cryosurgery ablation of a solid tumor can be viewed as a vaccine “auto-inoculation.” Cryosurgery releases hundreds of unique antigens from a heterogeneous population of tumor cells that make up the invading cancer (80). These antigens are likely not just surface epitopes, but perhaps represent more attractive intracellular and intranuclear antigens as well. Studies of autoimmune diseases indicate that intranuclear and organelle-derived antigens may be much more potent stimulators of the host’s immune system, processed by the innate and adaptive immune responses (20).

## Cryosurgery Promotes a CD8^+^ Cytotoxic Response to Cancer

Is there evidence that cryosurgery ablation of a solid tumor can result in stimulation of the host’s immune system yielding a favorable response directed against primary and metastatic tumors? The answer is a resounding yes, and its mechanisms have been well described in the literature under the abscopal effect. The abscopal effect (“ab”- away from,“scopus”- target) was coined in 1953 by Dr. R.H. Mole after his group observed a notable regression of distant disease two days after a primary tumor was irradiated (Figure 1) (81). While this phenomenon is infrequently observed, its effect on cancer can be striking, yielding such a robust response that causes malignant lesions to regress throughout the entire body. This achievement has been described in several cancers, including prostate cancer, renal cell carcinoma, melanoma, and cutaneous lymphomas (82).

Cryosurgery-induced tumor and cell death processing includes two separate zones of destruction. The inner zone of cryosurgery ablation results in tumor coagulation necrosis and is known as the central zone where cold temperatures reach −50°C (83). The antigens of this central area are characterized by cellular breakdown and release of intracellular contents, but may indeed be cell surface antigens as well as nuclear antigens (83). The central zone cytokine milieu resulting from cryosurgery is typically a T_H_1 cytokine profile of IL-2, IFN-γ, TNF-α, and IL-12 (84). These central zone antigens should then be processed *via* the MHC Class I leading to a cytotoxic T-cell response. The outer periphery zone of cryosurgery ablation results in cell death *via* apoptosis through extracellular freezing of fluid and osmosis, creating a shift of fluid from the intracellular to the extracellular microenvironment which pushes the immune response to the unfavorable T_H_2 cellular response (83). However, it has been determined that 7 days following cryosurgery, the T_H_1 response (anti-cancer) dominates the T_H_1/T_H_2 ratio and yields the desired antitumor effects (85). Surgical extirpation, on the other hand, causes a T_H_2 dominated immune response at the site of intervention (85). In addition to tumor cell destruction, cryosurgery also results in enhanced cellular immunity. Thawing causes further cell membrane disruption and death, a process called recrystallization and in which apoptosis is thought to predominate (83). These peripheral cryoablated cells will be processed by a MHC Class II resulting in an antigen-specific response. However, the biochemical effects of peripheral zone cell death can be highly variable from patient to patient (84).

Different cancer cell lines produce varying degrees of immune stimulation or suppression. The cytokine profile produced with cryosurgery in this outer zone is a T_H_2-cell response, which includes increased expression of IL-4, IL-5, and IL-10 (86). Regardless, cryosurgery apparently results in an immune response of cryo-self-antigens or iso-antigens that stimulate an antitumor response (87). Identifying these new circulating tumor-derived antigens and optimizing the targeted response in the host is advantageous and should result in regression of not only the tumor of origin but distant metastases as well (Figure 2). If a strong, robust dual immune response can be developed by the host, both future primary (local), regional and metastatic recurrences should be preventable by conferred immunity as well.

As hypothesized, several investigators have demonstrated that the cryosurgical abscopal effect has not only been observed but is mediated by cytotoxic CD8^+^ T-cells under the control of Tregs in both breast and prostate cancer models (53). Sabel et al. (84) demonstrated that mice with 4T1 breast xenografts which were cryoablated led to a significant decrease in distant pulmonary metastases and a prolonged survival advantage compared to control (surgery excision only). The ratio of CD4^+^ to CD4^+^CD25^+^ Treg cells as well as the level of cytokine release of IFN-γ was analyzed between low and high rates of freezing. These findings correlated an immunosuppressive increase in CD4^+^CD25^+^ Treg cells and low IFN-γ levels with the low rate of freeze (84). Conversely, a high rate of freeze lead to increased expression of IFN-gamma and a lower number of CD4^+^CD5^+^ Tregs which correlated with a desirable immune-profile, resulting in regression of distant pulmonary metastases (84).

To further support our hypothesis, in a study by Levy et al. (88), the investigators used cyclophosphamide to selectively deplete CD4^+^CD25^+^FoxP3^+^ Treg cells, allowing the T-effector CD8^+^ cytotoxic T-cells to evolve in response to cryoablation of a tumor. The cyclophosphamide plus cryosurgery group compared to surgery alone lived 125 days vs. 24 days. These cytotoxic CD8^+^ T-cells were then adoptively transferred into naïve Balb-c mice with the same established cancer and resulted in a significantly longer survival (>150 days vs. 56 days) (88). Finally, Waitz et al. (24) identified the CD4^+^CD25^+^FoxP3^+^ Treg suppressor cells responsible for suppressing the evolution of T effector cells in promoting a cytotoxic CD8^+^ T-cell response to cryoablation by administering CTLA-4 antibodies.

Altogether, these findings support our hypothesis that cryoablation results in a shower of tumor antigens released and presented to the host immune system. When the host’s immune system is “primed” with CTLA-4 blockade to favor a balanced ratio of Teff cells >Tregs, a cytotoxic CD8^+^ T-cell response will predominate, resulting in conferred immunity to the cancer and regression of not only the primary tumor but of its metastatic deposits as well.

## Cryosurgery Releases a Deluge of “Self-Antigens”

**Figure 3:**
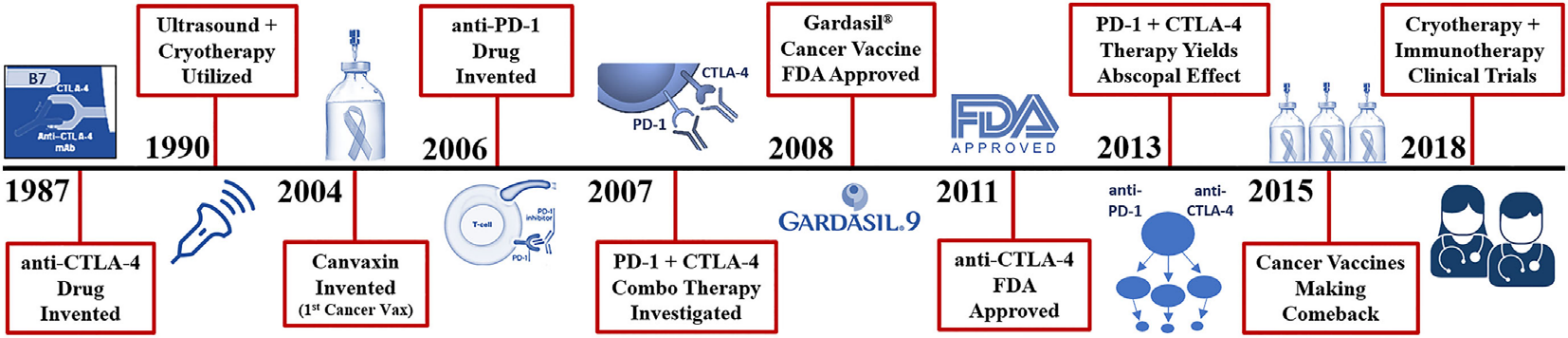
Recent history of cryosurgery and immunotherapy R&D. Vaccines are the only mechanism in which entire species of genome have been eradicated from the human population (smallpox, chickenpox, measles virus). Presently, cancer vaccines like Gardasil 9^®^ can reduce the number of cervical, and likely oral cancers observed through the protection from 9 types of inciting human papilloma viruses. True cancer vaccines, such as Canvaxin™ (2004), have been produced and investigated but most have yielded underwhelming results in creating an effective highly targeted tumor antigen which would likely kill a pure monoclonal or homogenous tumor yet fails in a heterogenous population of cancer cells. Many of the highly specific vaccines developed for melanoma, glioblastomas, and colon cancer have been ineffective or weakly effective; however, now when combined with cytotoxic T-lymphocyte-associated protein (CTLA-4) and PD-1 checkpoint inhibitors, are becoming more effective and garnering more clinical investigation. The addition of checkpoint inhibitors to potentiate these more advanced cancer vaccines does appear to yield impressive outcomes but still with lower than desired effects.

In 1967, Yantorno et al. (89) first identified and described how cryosurgical ablative intervention resulted in the production of antibodies directed at antigenic material linked to the frozen tissue itself (Figure 1). They reported that freezing male rabbit accessory tissue sensitized with glandular cellular components *via* injection, led to the formation of circulating antibodies (89). This autoinoculation resulted in a potentiated targeted antigenic response to the treated tissue which prompted the investigators to coin the term “iso-antigens” or self-antigens (89). The kinetics of these specific antigen-antibody complexes were analyzed, and the level of complexes reached its peak at day 7 or 8 following cryo-immunization (89). These findings suggest that freezing tissue at −196°C simulates the effects of an antigen-specific immune response (Figure 3). This was the first time the immune effects of cryoablation were elucidated and described, demonstrating how cryoablation could be utilized as an endogenous tumor vaccine and could lead to an observed abscopal effect.

Minimally invasive interventional ablative therapies are evolving rapidly, and their use for the treatment of solid tumors is becoming more extensive. *In situ* destruction of cancerous lesions *via* thermal and non-thermal ablative platforms has been found to produce tumor-specific antigens that can lead to a targeted antitumor immune reaction (Figure 2) (80). Benign and malignant tumors of all types have been frozen and destroyed by cryosurgery of various organs of origin including breast, prostate, colon, renal, pancreatic, esophageal, skin, brain, and lung (86). Cryosurgery has surgical ablative advantages due to minimal invasiveness and enhanced targeting and has demonstrated a high safety profile for patients. The literature is replete with successful ablation of various primary and metastatic tumors utilizing cryosurgery ablation and is an acceptable means to destroy such lesions compared to the gold standard of surgical excision (86). Arnott first froze breast tissue in 1851, and now cryosurgery is a well-accepted method of ablating and destroying both benign and malignant tumors (Figure 1) (3, 84).

Cryosurgery of breast malignancies has been performed in patients with co-morbidities who are unable to undergo standard surgical excision. Cryosurgery can also be used as a means of anesthesia and, therefore, has become an office-based procedure for the destruction of skin and breast cancers and benign fibroadenomas. The Phase II trial ACOCOG Z1072 was a multi-institutional trial safely demonstrating an effective means of ablating invasive breast cancers (16). As aforementioned, the FACT, FROST, ECLIPSE, and SOLSTICE studies and other additional human trials of cryoablation in benign and early stage/resectable breast, lung, and esophageal cancers are in progress (Table 1) (9). Many recent investigations, presented herein, also considered the immunogenic effects of cryoablation in different cancer types.

Investigating alterations in cellular immunity following argon-helium cryoablation, Li et al. (85) designed an experiment with rats exhibiting subcutaneous gliomas and divided them into normal control (*n* = 30), sham-operated (*n* = 33), surgical resection (*n* = 30), and cryosurgery (*n* = 33) groups. The surgical resection group exhibited a substantial decrease in the cell percentages of CD3^+^ and CD4^+^ lymphocytes, CD14^+^ monocytes, and CD16^+^CD56^+^ natural killer cells, where the cryoablation group had significant amplification of the same cell subpopulations while also exhibiting an increased T_H_1/T_H_2 ratio seven days post cryoablation (85). These outcomes establish that cryosurgery results in improved cellular immunity, while also demonstrating more effective tumor destruction—highlighting the clinical utility of cryosurgery in the management of solid tumors.

A subcutaneous xenograft rat tumor model directly evaluated the immune response as a result of cryotherapy by measuring cytokine expression levels, and analyzing T-cell responses to tumor-derived antigens (87). In this study, cryoablation not only triggered tumor destruction but also induced apoptosis near the cryosurgery foci. Surgical resection yielded a noteworthy decrease in CD3^+^ and CD4^+^ cells indicating a reduction in T-cell production, while cryosurgery caused a significant amplification of CD3^+^ and CD4^+^ cells with an improved CD4^+^/CD8^+^ cell ratio demonstrating a more aggressive T lymphocytic response to pathogens (87). Furthermore, cytotoxicity of mononuclear cells was significantly augmented in the cryoablation group. Yet again, a comprehensive dataset validates that cryosurgery influences the immune system to optimize cellular immunity against carcinogenesis while also facilitating a targeted antitumor immune response after complete destruction of the tumor, suggesting the vast potential of cryoablation to induce a robust abscopal effect in late stage cancers.

Kim et al. (90) assessed immunologic responses and subsequent effect of cancer ablation with cryotherapy compared to surgical excision in a kidney tumor murine model. Mice growing xenograft kidney tumors underwent either cryoablation or surgical excision. The mice that experienced a progression-free response after the first intervention were re-challenged with either the same renal cancer cell line or a cell line derived from a colon cancer (CT26) to determine if the observed immune response was tumor specific (90). First, the rate of tumor regrowth occurred more rapidly and in a significant number of more animals in the surgical excision group compared to the cryoablation group (94.4 vs. 11.1%) (90). The cryoablation group also demonstrated elevated CD4^+^ and CD8^+^ T-cell counts as well as an increased number of natural killer cells, while also demonstrating a significant increase of cytotoxicity in comparison to the surgery-only cohort (90). Cryoablation was decidedly more effective than surgical resection in preventing tumor growth after rechallenge with the same syngeneic renal cancer cells. Investigators also deemed the immune response to be tumor-specific because tumor regression occurred upon rechallenge with this syngeneic kidney cancer while tumor progression occurred with the colon cancer rechallenge—denoting a targeted anti-cancer immune response in the cryoablation group.

Radiotherapy and cryotherapy research is replete with an abundance of evidence for initiating and stimulating an immuno-modulatory response against many cancers (Tables 1 and 2) (82, 86). The immune response to autoinoculation by tumor destruction and the subsequent “shower” of self-antigens may be significantly heightened by merging these platforms with other immunostimulatory therapies. The immunomodulatory consequence linked to thermal and non-thermal ablative therapies utilized in medical oncology cases is amassing rapidly (80). Additionally, the mechanism behind the resultant abscopal effect is beginning to be elucidated and hopefully will lead to the optimization of this new paradigm as an effective anti-cancer strategy. Klarquist et al. (91) attributes the STING pathway as being one of the main promoters of adaptive immune antitumor responses. These findings demonstrate that the abscopal effect does not require toll-like receptors or NOD-like receptors to trigger programmed cell-death in cancer tissue but requires STING exclusively when initiating a targeted CD8^+^ T-cell anti-cancer response (91). Regardless, future research investigating these various modalities will be required in animal models and prospective randomized multi-center human trials.

## Immune System Manipulation to Elicit Tumor Regression

The rudiments of immunotherapy were first discovered in 1891 when Dr. William Coley witnessed the power of the immune system in anti-cancer therapy when a patient with head and neck sarcoma went into full remission after his body fought a streptococcus infection which occurred at the site of surgery (Figure 1) (92). The more his patient fought the infection, as evidenced by high fevers to fend off the unrelated contagion, the more the facial tumor regressed until the patient was in complete remission. This phenomenon was discovered over 125 years ago, yet the human’s own immune defense system has remained obscure and almost completely ignored in the fight against cancer for centuries. Interestingly, immunostimulation with Coley’s “toxins” reported a near 40% regression of tumors in some series (93). Freund’s complete adjuvant is an effective immune modulator similar to Coley’s toxins which stimulates cell-mediated immunity and yields an augmentation of T helper cells with the production of targeted immunoglobulins and effector T-cells (94). However, with the recent entrance of the newest arsenal in the war on Cancer: anti- CTLA-4 and PD-1 checkpoint inhibitors have now taken the molecular oncology arena by storm (Figure 3). The immune system has evolved over many millennia and has become the perfection of natural selection, albeit deceived by the cancers’ evasive maneuvers such as local tumor microenvironment and systemic escape mechanisms (e.g., PD-L1 expression). These local and systemic cancer escape mechanisms have been well described throughout the literature (95).

Manipulation of the immune system has been pursued over the past century to conquer cancer. While some attempts were successful in causing regression of cancers and prolonging disease-free and overall survival, others were not. One well-described method of immune manipulation is the injection of tetanus toxoid or BCG (96, 97). High dose (HD) IL-2 is another well-described immunotherapy that has been administered in both melanoma and renal cell carcinoma with a complete and durable response in approximately 8% of patients (98).

Not only is the use of IL-2 a remarkable manipulation of the host’s immune system resulting in eradication of the invading cancer, but it has also demonstrated one of the most durable long-term responses in cancer treatment. SELECT and PROCLAIM are IL-2 patient databases with more than 40 participating sites consisting of retrospective melanoma and prospective melanoma cohorts (99, 100). The registry is designed to collect data from community oncologists and large academic centers on the use of HD IL-2 in the treatment of metastatic renal cell carcinoma or metastatic melanoma (99). These therapies favor a T_H_1cytotoxic T-cell response with the associated cytokine profile.

IL-12 is yet another well-described interleukin with favorable antitumor immune activity. There are at least 25 active clinical trials revisiting the effects of IL-12 on cancer (101). What is common to these immune adjuvants is they reflect a shift toward an MHC Class I cytotoxic T-cell response and often involve crosstalk between the innate and adaptive immune systems, which is critical in achieving an abscopal effect in stage III and IV cancer patients.

This dual response is known as cross-presentation and is the process by which exogenous antigens, typically processed by APCs and MHC Class II cells, can cross over and be presented to the MHC Class I pathway to generate a cytotoxic t-cell response (102). Endogenous antigens, on the other hand, from self-cancer cells or virus infected cells, are processed by the MHC Class I pathway, again resulting in cytotoxic T-cells (103). However, this cross-presentation is a dual mechanism, as the MHC Class I – CD8^+^ pathway likely can be presented and crossover to the MHC Class II—CD4^+^ process, again resulting in a dual crosstalk (102). The effector cells or Tregs managing this process are crucial to the outcome of this complex immune response. Again, we believe the ratio of Tregs to T-effector cells and their cytokine profile dictate and control this homeostatic “set-point” and when skewed to a T_C_1 cytotoxic CD8^+^ T-cell and associated cytokine profile, the host’s immune response will prevail, and the invading cancer will be eradicated. Therefore, the way cryoablated tumor antigens are processed by this orchestrated immune response will dictate whether the final immune response is stimulatory and efficacious or inhibitory and not therapeutic.

## Enhanced Abscopal Effect with Immunotherapy Primer

Radiation therapy, recognized for its potent cytotoxic effect on cancer cells by inducing direct DNA damage, can sometimes elicit a systemic antitumoral response. Irradiation releases a plethora of neoantigens and pro-inflammatory cytokines, acting like an *in situ* vaccine, resulting in tumor regression within the primary site, but may also occasionally result in regression of distant secondary lesions (47). This regression of distant cancer metastases when the primary tumor is irradiated is defined as the abscopal effect. Yet, an abscopal effect with radiotherapy alone occurs infrequently, signifying that the antitumor immunity caused by radiation is not sufficient enough to abolish the tumor and its metastases nor able to prevent the metastatic process or the immunosuppressing effect the cancer exhibits on the host’s systemic macroenvironment. Recently, several studies have confirmed the synergistic antitumoral immunity caused by the combination of radiation with immunotherapy, which has demonstrated a durable abscopal effect in patients with advanced malignancies (Table 2). Postow, et al, Golden, et al, Hinicker, et al and others have all described early findings of a reproducible abscopal effect when combining irradiation with Ipilimumab and/or Nivolumab (44, 47, 48).

Mesothelioma is an aggressive thoracic neoplasm with a median overall survival less than 1 year following standard cancer treatment consisting of combination chemotherapy (1). Because of the underwhelming performance of chemotherapy, there has been an increased interest in utilizing immunotherapy as first-line and salvage treatment options. The leading class of immunotherapies investigated in mesothelioma are the immune checkpoint inhibitors. Preliminary data has been encouraging, mainly for drugs targeting the PD-1/PD-L1 pathway. There is now growing recognition of the immune system’s potential to eradicate mesothelioma, such that research into the immunomodulatory effects of radiation has become a rapidly emerging field of study (104). The combination of immunotherapy and radiation therapy is yielding evidence of complimentary immunomodulatory forces that can augment antitumor responses (104). However, we propose a safer, less toxic, and more efficient avenue for autoinoculation would be the utilization of cryoablation following immunotherapy (Figure 2).

Expanding on the findings of Klarquist et al. (91), a 2017 study analyzed if anti-CTLA-4 and anti-PD-1 therapy used in combination with myeloid agonists targeting STING or Flt3 could control multifocal disease in a prostate cancer xenograft model (105). STING agonists improved the therapeutic effect of combination checkpoint modulation and promoted an abscopal effect in 75% of mice (105). Double T-cell checkpoint modulation correlated with greater ratios of CD8^+^ T-cells to Treg cells, macrophages, and myeloid-derived suppressor cells which in turn produced impressive antitumor activity (105).

IL-12 is considered one of the most effective immunostimulatory cytokines and is a complimentary fusion protein of IL-12’s functional domain. IL-12 therapy has demonstrated significant terminal growth arrest against human rhabdomyosarcoma xenografts in a humanized tumor model (106). Another 2017 study utilizing anti-IL-12 demonstrated that locally irradiated tumors exhibited an increase in both tumor necrosis and intratumoral immune cytokine activity. Eckert et al.(106) also considered whether this effect might surmount efficacy of a single treatment modality. Humanized mice bearing bilateral rhabdomyosarcoma xenografts were evaluated for tumor burden and survival after radiation alone, systemic IL-12 therapy alone or a combination of both. Data revealed that when IL-12 treatment was administered in combination with radiation the subjects experienced significant tumor regression and extended overall survival (106). Dual therapy also yielded a thick cluster of intratumoral T-cells at the site of carcinoma, an increased expression of various antitumor cytotoxins, increased epitope-specific T-cell production, reduced pro-tumorigenic cytokines and generated more robust senescence in proliferating cells (106). Combination of IL-12 and radiation induced extensive intratumoral NK and T-cell propagation while causing increased expression of antitumoral cytokines which irreversibly arrested the growth of tumor cells (106). This combination treatment modality created a perfect storm which leads to systemic control of cancer progression and improved survival. Here, we have a model for immune-induced tumor destruction as a novel mechanism resulting from a treatment regimen combining radiation with immunotherapy.

To this, a case study from 2017 showed resounding results as a 48-year-old male with squamous non-small cell lung carcinoma did not respond well to first-line chemotherapy or second line anti-PD-1 immunotherapy (107). Approximately 15–20% of unselected advanced cancer patients mount a significant response to immunotherapy with PD-1 immune-checkpoint inhibitors (108). As a last resort to extend survival, the patient was given 10 fractions of palliative radiation. Post radiation, the patient developed global response after the immune system was “primed” with 3 weeks of anti-PD-1 treatment (107). This patient continues to have progression-free survival and is off all anti-cancer treatment with follow up CT scans every 2–3 months (107). Although this case is anecdotal, the fact that anti-PD-1 therapy followed by radiation essentially rescued a patient from refractory status is quite remarkable and encouraging.

## Cryotherapy and Immunotherapy can Induce an Abscopal Effect

Immunotherapy is revolutionizing the systemic management of many malignancies (28). Numerous preclinical and clinical studies have revealed the potential benefit of immune-priming radiotherapy in stimulating tumor-specific immune responses (53). Immune activation *via* primary ablation/radiation is showing signs of clinically-relevant synergy with immune checkpoint inhibitors against malignant tumors (Table 2). Very few studies have analyzed if cryoablation of a primary tumor combined with immunotherapy can yield regression of distant metastases.

An animal model experiment explored variations in antitumor immunity of rats with glioblastoma xenografts that were treated with argon–helium cryoablation combined with IL-12 treatment (109). In this study, rats were separated into various treatment groups (IL-12 therapy alone vs. cryotherapy alone vs. cryotherapy plus IL-12 therapy vs. control). The cryotherapy alone and cryotherapy plus IL-12 therapy groups both revealed a significant increase in cellular immunity, with the cryotherapy plus IL-12 therapy group having by far the most notable increases in tumor regression and subsequent hyper-immune activity (109). In the cryotherapy group, tumors were destroyed, and an increase of APCs was noted. The quantity of CD11c^+^ dendritic cells and CD86^+^ lymphocytes significantly increased post-cryoablation. Interferon-γ levels were also augmented, signifying a change to T_H_1-type immunity. In this rat glioblastoma model, the combination therapy group (cryoablation plus immunotherapy) experienced complete disintegration of their solid tumors with enhanced immune function and increased antitumor ability (109). But, would cryotherapy preceded by immunotherapy exhibit clinical utility in a human clinical trial? Unfortunately, there has been limited investigation in this arena on human patients; however, a recent clinical trial in pancreatic cancer patients has shown some encouraging results.

Pancreatic carcinoma is a disease with increasing incidence and has one of the worst prognoses in oncology (1). Most patients present in late stages, thus eliminating them from a possible curative surgery option. Cryoablation is deemed an effective palliative therapy for advanced pancreatic carcinoma and is often suitable for patients with unresectable pancreatic cancer. However, in the only clinical trial of its kind, Niu et al. (110) suggest that cryotherapy combined with immunotherapy for advanced pancreatic cancer can significantly extend survival in patients with metastatic pancreatic cancer. Subjects were divided into the following treatment groups: cryo-immunotherapy, cryotherapy, immunotherapy, and chemotherapy. The results showed that the median overall survival periods in the cryo-immunotherapy and cryotherapy groups (13 months and 7 months) were significantly longer than those in the chemotherapy group (3.5 months) (110). The median overall survival time in the cryo-immunotherapy group was also significantly prolonged compared to each monotherapy group (110). Thus, longer survival durations could be achieved by either cryo-immunotherapy or to a lesser extent, cryotherapy alone—particularly when several cryoablation applications are administered compared to a single procedure.

Although there is a paucity of literature and clinical data investigating this therapy model in humans, there are a few emerging and notable studies demonstrating that immunostimulation followed by cryotherapy can shrink distant metastases and prolong overall survival in stage IV cancer patients. Cryotherapy in conjunction with granulocyte-macrophage colony-stimulating factor, which promotes dendritic cell activation, has demonstrated shrinkage of metastases in lung cancer patients (111). As mentioned above, the same combination in pancreatic patients was seen to increase overall survival (110). Nevertheless, additional clinical trials are needed to verify if this method will not only result in the destruction of the primary lesion but will also cause regression of distant tumor metastases.

## Expert Commentary and Future Directions

Cancer is the number two killer of human lives in America and worldwide and is expected to surpass atherosclerosis and heart disease as the number one cause of death on the planet (112). Last year Joe Biden, former Vice President of the United States declared a “War on Cancer” in his “2020 Moonshot” initiative (113). This is not the first time the American government has issued a call to arms against cancer, as former President Richard Nixon also declared a war on cancer with the National Cancer Act of 1971 with the aim to “cure” cancer within 5 years (114). It is a critical time to investigate novel methods to markedly improve patient management strategies. Utilizing the powerful and dynamic potential of the immune system refined by the natural selection process over many millennia, anti-cancer immunotherapy regimens are now being held with increased importance in the oncology arena.

One of the most effective immune stimulating methods to completely eradicate a human disease is a vaccine. Recognizing that cryosurgery and, to a lesser degree, radiation therapy both produce and release numerous *in situ*, tumor-specific, self-antigens and epitopes from a heterogeneous population of cancer cells into the host’s systemic circulation, is analogous to a polyvalent, auto-inoculation of tumor self-antigens, and can be considered an auto-vaccine, led to formation of our hypothesis. Capitalizing on the phenomenon of a radiation or cryosurgery-induced abscopal effect, now being observed at a remarkable rate in both animal and human subjects with the addition of immunotherapy or checkpoint inhibitors, has substantiated further development of our hypothesis.

Cryoablation or radiation preceded by immunotherapy has demonstrated an abscopal effect in both animal models and humans. The combination of anti-CTLA-4 and/or anti-PD-1 immunotherapy potentiates the immune response to cryoablated primary tumors. When the host’s immune system has been pre-treated and “primed” with the combined immune adjuvants anti-CTLA-4 and/or anti-PD-1 antibodies, these preserved cryoablated tumor antigens will be presented to and processed by the hosts immune system predisposed to a higher ratio of T-effector cells/Treg suppressor cells resulting in a robust cytotoxic CD8^+^ T-cell response (86). This form of targeted autoinoculation should produce a comprehensive abscopal effect with complete regression of both the primary tumor and metastases as well as preventing recurrences, either locally or distantly through conferred immunity, thus extending disease-free survival and overall survival.

The choice and duration of the immunotherapy regimen a patient is placed on is determined by the patients’ oncologist or clinical trial protocol. However, based on past clinical trials and preclinical animal models (Table 2), we suppose that two to four doses of combination immunotherapy prior to cryoablation would be a sufficient primer to inhibit cell death checkpoints and allow a deadly cytotoxic immune response to evolve. Postow et al. (48) were able to achieve a significant abscopal effect with four doses of a single anti-CTLA-4 drug as part of the induction therapy prior to radiation. The patient observed in this case study showed no evidence disease even 12 weeks after immunotherapy had ceased (48). Furthermore, given the data that cryo-antigens have been observed up to several weeks following tumor ablation cryosurgery, we suppose that two to four additional combined doses of CTLA-4 and PD-1 inhibitors be administered post-cryosurgery as well. Cryosurgery has the potential to produce thousands of self-antigens which are released into circulation for days and even weeks following cryo-ablative surgery. In essence, an *in situ*, poly-valent, autoinoculation vaccine of many cancer-specific antigens, derived from a heterogeneous population of tumor cancer cells, would be presented to the host’s unhindered yet primed and sensitized immune system, yielding a superior advantage in locating, recognizing, targeting, and destroying tumor cells throughout the body. These released self-antigens should indeed be processed by the immune system if the “programmed” T-cell checkpoint escape mechanisms are blocked, thereby priming the patient’s immune system prior to direct contact with these cryoablated and presented self-antigens. This will allow both the tumor antigens and the host’s immune system to circumvent the cancer’s defense mechanisms (36). Therefore, administering anti-CTLA-4 and anti-PD-1 therapies in a neoadjuvant setting prior to cryosurgery is paramount to the success of this proposed paradigm. Whether or not patients should continue maintenance immunotherapy after an abscopal effect has been achieved will hopefully be answered with formal clinical trials.

Herein, we propose a new paradigm shift on the war against cancer by utilizing the host’s own immune defense mechanisms *via* ablative *in situ* destruction of the primary tumor preceded by immune stimulation with CTLA-4 and PD-1 checkpoint inhibitors. Based on recent investigations and well-described biochemical mechanisms presented, we propose that immunotherapy followed by cryoablation of the primary tumor would result in a profound synergistic abscopal effect (Figure 2). We have provided evidence of this phenomenon that gives hope to patients battling late stage or aggressive cancers as well as to those patients whom may develop recurrences. When immunotherapy and cryoablation are combined sequentially, we would anticipate the patients’ immune response will be far more effective in eradicating the patient’s cancer compared to chemotherapy, radiation, immunotherapy, or surgical extirpation alone. Regardless, additional clinical investigations into this new therapeutic platform are certainly warranted.

## Author Contributions

DC brought these clinical concepts and sequences to our group. JA and DC wrote the original draft. JA and DA designed the figures. All four authors revised and refined multiple drafts of the review and various versions of the figures.

## Conflict of Interest Statement

The authors have no other relevant affiliations or financial involvement with any organization or entity with financial interest or financial conflict with the subject matter or materials discussed in the manuscript apart from those disclosed. No writing assistance was utilized in the production of this manuscript.
